# Supplementary material for: STRIPAK directs PP2A activity toward MAP4K4 to promote oncogenic transformation of human cells
Source: eLife. 2020 Jan 8;9:e53003. doi: 10.7554/eLife.53003 (PMC6984821; doi:10.7554/eLife.53003)
Supplement: Supplementary file 1. [file elife-53003-supp1.docx]

| **Key Resources Table** | | | | |
| --- | --- | --- | --- | --- |
| **Reagent type (species) or resource** | **Designation** | **Source or reference** | **Identifiers** | **Additional information** |
| **Antibody** | Rabbit monoclonal anti-HA-Tag (C29F4) | Cell Signaling Technology | Cat# 3724  RRID:AB_1549585 |  |
| **Antibody** | Rabbit monoclonal anti-GFP (D5.1) | Cell Signaling Technology | Cat# 2956S  RRID:AB_1196615 |  |
| **Antibody** | Mouse monoclonal anti-vinculin (H-10) | Santa Cruz Biotechnology | Cat# sc-25336  RRID:AB_628438 |  |
| **Antibody** | Rabbit monoclonal anti-β-Actin (D6A8) | Cell Signaling Technology | Cat# 8457  RRID:AB_10950489 |  |
| **Antibody** | Rabbit monoclonal anti-thiophosphate ester (TPE) | Abcam | Cat# ab92570  RRID:AB_10562142 |  |
| **Antibody** | Rabbit polyclonal anti-PPP2R1A | Bethyl Laboratories, Inc. | Cat# A300-962A  RRID:AB_805812 |  |
| **Antibody** | Rabbit polyclonal anti-PP2A C | Cell Signaling Technology | Cat# 2038  RRID:AB_2169495 |  |
| **Antibody** | Rabbit polyclonal anti-STRN3 | Thermo Fisher Scientific | Cat# PA5-31368  RRID:AB_2548842 |  |
| **Antibody** | Rabbit polyclonal anti-MAP4K4 (HGK) | Cell Signaling Technology | Cat# 3485S  RRID:AB_2140972 |  |
| **Antibody** | Rabbit polyclonal anti-STRIP1 (FAM40A) | Thermo Fisher Scientific | Cat# PA5-26722  RRID:AB_2544222 |  |
| **Antibody** | Goat polyclonal anti-CTTNBP2NL (P-12) | Santa Cruz Biotechnology | Cat# sc-137409  RRID:AB_10838519 |  |
| **Antibody** | Rabbit polyclonal anti-SV40 T antigen (V-300) | Santa Cruz Biotechnology | Cat# sc-20800  RRID:AB_661493 |  |
| **Antibody** | Rabbit polyclonal anti-YAP | Cell Signaling Technology | Cat# 4912  RRID:AB_2218911 |  |
| **Antibody** | Rabbit polyclonal anti-phospho-YAP (S127) | Cell Signaling Technology | Cat# 4911S  RRID:AB_2218913 |  |
| **Antibody** | Rabbit polyclonal anti-LATS1 | Bethyl Laboratories, Inc. | Cat# A300-477A  RRID:AB_451012 |  |
| **Antibody** | Rabbit monoclonal anti-phospho-LATS1 (T1079) (D57D3) | Cell Signaling Technology | Cat# 8654S  RRID:AB_10971635 |  |
| **Antibody** | Goat polyclonal anti-CTGF (L-20) | Santa Cruz Biotechnology | Cat# sc-14939 |  |
| **Antibody** | Rabbit polyclonal anti-Cyr61 (H-78) | Santa Cruz Biotechnology | Cat# sc-13100  RRID:AB_2088733 |  |
| **Antibody** | Rabbit monoclonal anti-STRN4 (EPR11801) | Abcam | Cat#: ab177155 |  |
| **Chemical compound, drug** | MAP4K4 inhibitor (compound 29) | (Crawford et al. 2014) | N/A |  |
| **Chemical compound, drug** | Protease and phosphatase inhibitor cocktail sets | Calbiochem | Cat# 524624  RRID:AB_524624 |  |
| **Chemical compound, drug** | Laemmli (SDS-Sample Buffer, Reducing, 6X) | Boston BioProducts | Cat# BP-111R |  |
| **Commercial assay, kit** | Clarity Western ECL substrate | (Bio-Rad) | Cat# 1705060 |  |
| **Commercial assay, kit** | Pierce™ Protein A Agarose | Thermo Scientific | Cat# 20333 |  |
| **Chemical compound, drug** | Anti-FLAG M2 Magnetic Beads | Sigma | Cat# M8823 |  |
| **Peptide, recombinant protein** | HA peptide | Roche | Cat# 11666975001 |  |
| **Peptide, recombinant protein** | Flag peptide | Sigma | Cat# F3290 |  |
| **Commercial assay, kit** | SILAC Protein Quantitation Kit (LysC), DMEM | Thermo Scientific | Cat# A33969 |  |
| **Chemical compound, drug** | ATPγS | Abcam | Cat# ab138911 |  |
| **Chemical compound, drug** | P-nitrobenzyl mesylate (PNBM) | Abcam | Cat# ab138910 |  |
| **Chemical compound, drug** | Dimethyl sulfoxide (DMSO) | Sigma | Cat# D4540 |  |
| **Chemical compound, drug** | Lipofectamine 2000 | Life Technologies | Cat# 11668019 |  |
| **Chemical compound, drug** | Polybrene | Santa Cruz | Cat# sc-134220 |  |
| **Chemical compound, drug** | Puromycin | Sigma | Cat# P8833 |  |
| **Chemical compound, drug** | Blasticidin | Invivogen | Cat# ant-bl-05 |  |
| **Chemical compound, drug** | Neomycin | Sigma | Cat# N1142 |  |
| **Chemical compound, drug** | Hygromycin | Santa Cruz | Cat# H3274 |  |
| **Commercial assay, kit** | PP2A Immunoprecipitation Phosphatase Assay Kit | Millipore Sigma | Cat# 17-313 |  |
| **Commercial assay, kit** | Silver staining | GE Healthcare | Cat# 17-1150-01 |  |
| **Commercial assay, kit** | QuikChange XL II site-directed mutagenesis kit | Agilent | Cat# #200521 |  |
| **Commercial assay, kit** | Gateway™ BP Clonase™ II Enzyme mix | Invitrogen | Cat# 11789020 |  |
| **Commercial assay, kit** | Gateway™ LR Clonase™ II Enzyme mix | Invitrogen | Cat# 11791100 |  |
| **Recombinant DNA reagent** | lentiCRISPRv2 | Addgene | 52961 |  |
| **Recombinant DNA reagent** | MSCV-N-terminal-Flag-HA-IRES-PURO (NTAP) | Addgene | 41033 |  |
| **Recombinant DNA reagent** | MSCV-C-terminal-Flag-HA-IRES-PURO (CTAP) | Berrios et al. 2015 |  |  |
| **Recombinant DNA reagent** | MSCV-C-terminal-Flag-HA-IRES-PURO (CTAP)- SV40 ST | This study |  |  |
| **Recombinant DNA reagent** | MSCV-C-terminal-Flag-HA-IRES-PURO (CTAP)- JCV-CY ST | This study |  |  |
| **Recombinant DNA reagent** | MSCV-C-terminal-Flag-HA-IRES-PURO (CTAP)- JCV-mad1 ST | This study |  |  |
| **Recombinant DNA reagent** | MSCV-C-terminal-Flag-HA-IRES-PURO (CTAP)-BKV ST | This study |  |  |
| **Recombinant DNA reagent** | MSCV-C-terminal-Flag-HA-IRES-PURO (CTAP)- SV40 ST-R21A | This study |  |  |
| **Recombinant DNA reagent** | MSCV-C-terminal-Flag-HA-IRES-PURO (CTAP)- SV40 ST-W147A | This study |  |  |
| **Recombinant DNA reagent** | MSCV-C-terminal-Flag-HA-IRES-PURO (CTAP)- SV40 ST-F148A | This study |  |  |
| **Recombinant DNA reagent** | MSCV-C-terminal-Flag-HA-IRES-PURO (CTAP)- SV40 ST-P132A | This study |  |  |
| **Recombinant DNA reagent** | pMSCV puro vector | Clontech | Cat# 634401 |  |
| **Recombinant DNA reagent** | pMSCV puro YAP1 WT | Lee et al. 2016 |  |  |
| **Recombinant DNA reagent** | pMSCV puro YAP1 5SA | Lee et al. 2016 |  |  |
| **Recombinant DNA reagent** | pBabe-hygro-hTERT | Addgene | Cat# 1773  RRID:Addgene_1773 |  |
| **Recombinant DNA reagent** | pBabe-HcRed-Ras | Addgene | Cat # 10678  RRID:Addgene_10678 |  |
| **Recombinant DNA reagent** | pBabe-neo- SV40 large T cDNA | Addgene | Cat # 1780  RRID:Addgene_1780 |  |
| **Recombinant DNA reagent** | pWZL-Blast-SV40 ST | Addgene | Cat # 13805  RRID:Addgene_13805 |  |
| **Recombinant DNA reagent** | pHCMV-AmphoEnv | Addgene | Cat # 15799  RRID:Addgene_15799 |  |
| **Recombinant DNA reagent** | pUMVC3 | Addgene | Cat # 8449  RRID:Addgene_8449 |  |
| **Recombinant DNA reagent** | psPAX2 | Addgene | Cat #12260  RRID:Addgene_12260 |  |
| **Recombinant DNA reagent** | pMD2.G | Addgene | Cat #12259  RRID:Addgene_12259 |  |
| **Recombinant DNA reagent** | MSCV-N-terminal-Flag-HA-IRES-PURO (NTAP)-MAP4K4-WT | This study |  |  |
| **Recombinant DNA reagent** | MSCV-N-terminal-Flag-HA-IRES-PURO (NTAP)-MAP4K4-K54R | This study |  |  |
| **Recombinant DNA reagent** | MSCV-N-terminal-Flag-HA-IRES-PURO (NTAP)-SV40 ST | Rozenblatt-Rosen et al. 2012 |  |  |
| **Recombinant DNA reagent** | MSCV-N-terminal-Flag-HA-IRES-PURO (NTAP)-GFP | Rozenblatt-Rosen et al. 2012 |  |  |
| **Recombinant DNA reagent** | pLKO_shRNA_STRN3-62 | Broad Institute Genetics Perturbation Platform | TRCN0000365162 |  |
| **Recombinant DNA reagent** | pLKO_shRNA_STRN3-06 | Broad Institute Genetics Perturbation Platform | TRCN0000370206 |  |
| **Recombinant DNA reagent** | pLKO_shRNA_STRN4-54 | Broad Institute Genetics Perturbation Platform | TRCN0000036954 |  |
| **Recombinant DNA reagent** | pLKO_shRNA_STRN4-55 | Broad Institute Genetics Perturbation Platform | TRCN0000036955 |  |
| **Recombinant DNA reagent** | pLKO_shRNA_STRN4-57 | Broad Institute Genetics Perturbation Platform | TRCN0000036957 |  |
| **Recombinant DNA reagent** | pLKO_shRNA_STRN4-58 | Broad Institute Genetics Perturbation Platform | TRCN0000036958 |  |
| **Recombinant DNA reagent** | pLKO_shRNA_STRIP1-02 | Broad Institute Genetics Perturbation Platform | TRCN0000164502 |  |
| **Recombinant DNA reagent** | pLKO_shRNA_STRIP1-51 | Broad Institute Genetics Perturbation Platform | TRCN0000162951 |  |
| **Recombinant DNA reagent** | pLKO_shRNA_MARCKS-45 | Broad Institute Genetics Perturbation Platform | TRCN0000197145 |  |
| **Recombinant DNA reagent** | pLKO_shRNA_ MARCKS-41 | Broad Institute Genetics Perturbation Platform | TRCN0000029041 |  |
| **Recombinant DNA reagent** | pLKO_shRNA_STK24-41 | Broad Institute Genetics Perturbation Platform | TRCN0000000641 |  |
| **Recombinant DNA reagent** | pLKO_shRNA_STK24-44 | Broad Institute Genetics Perturbation Platform | TRCN0000000644 |  |
| **Recombinant DNA reagent** | pLX304-STRN4-58R | This study |  |  |
| **Recombinant DNA reagent** | pLKO_shRNA_MAP4K4-92 | Broad Institute Genetics Perturbation Platform | TRCN0000220092 |  |
| **Recombinant DNA reagent** | pLKO_shRNA_MAP4K4-93 | Broad Institute Genetics Perturbation Platform | TRCN0000220093 |  |
| **Recombinant DNA reagent** | pLKO_shRNA_MAP4K4-58 | Broad Institute Genetics Perturbation Platform | TRCN0000195258 |  |
| **Recombinant DNA reagent** | pLKO_shRNA_MAP4K4-81 | Broad Institute Genetics Perturbation Platform | TRCN0000219681 |  |
| **Recombinant DNA reagent** | pLKO_shRNA_MAP4K4-82 | Broad Institute Genetics Perturbation Platform | TRCN0000219682 |  |
| **Recombinant DNA reagent** | pLKO_shRNA_MAP4K4-21 | Broad Institute Genetics Perturbation Platform | TRCN0000195121 |  |
| **Recombinant DNA reagent** | pLKO_shRNA_MAP4K4-25 | Broad Institute Genetics Perturbation Platform | TRCN0000199325 |  |
| **Recombinant DNA reagent** | pLKO_shRNA_YAP1 | Broad Institute Genetics Perturbation Platform | TRCN0000107265 |  |
| **Recombinant DNA reagent** | pLKO_shRNA_PP2A _Cα1 | Broad Institute Genetics Perturbation Platform | TRCN0000002483 |  |
| **Recombinant DNA reagent** | pLKO_shRNA_PP2A _Cα2 | Broad Institute Genetics Perturbation Platform | TRCN0000002484 |  |
| **Recombinant DNA reagent** | pLKO_shRNA_PP2A _ B56γ1 | Broad Institute Genetics Perturbation Platform | TRCN0000002494 |  |
| **Recombinant DNA reagent** | pLKO_shRNA_PP2A _ B56γ2 | Broad Institute Genetics Perturbation Platform | TRCN0000002496 |  |
| **Recombinant DNA reagent** | pLKO_shRNA_PP2A _ Aα | Broad Institute Genetics Perturbation Platform | TRCN0000231508 |  |
| **Recombinant DNA reagent** | lentiCRISPRv2-STRN4-gRNA1 | This study | TTGTGGAAAGGACGAAACACCcaccgCCAGGGTGACCGATTCCACGGTTTTAGAGCTAGAAATAGCA |  |
| **Recombinant DNA reagent** | lentiCRISPRv2-STRN4-gRNA2 | This study | TTGTGGAAAGGACGAAACACCcaccgTTTGGGACAGACCTGAACCAGTTTTAGAGCTAGAAATAGCA |  |
| **Recombinant DNA reagent** | lentiCRISPRv2-STRN4-gRNA3 | This study | TTGTGGAAAGGACGAAACACCcaccGGGCCCGCGCCTGAGCCGAGGTTTTAGAGCTAGAAATAGCA |  |
| **Recombinant DNA reagent** | lentiCRISPRv2-STRN4-gRNA4 | This study | TTGTGGAAAGGACGAAACACCcaccgCGAGGACGAAGACAGCGACGGTTTTAGAGCTAGAAATAGCA |  |
| **Software, algorithm** | ssGSEA | https://software.broadinstitute.org/cancer/software/genepattern/ |  |  |
| **Software, algorithm** | Morpheus | https://software.broadinstitute.org/morpheus/ |  |  |
| **Software, algorithm** | REVEALER | https://software.broadinstitute.org/cancer/software/genepattern/ |  |  |
| **Cell line** | HEK TER | (Hahn et al. 2002) |  |  |
| **Cell line** | HEK TER ST | (Hahn et al. 2002) |  |  |
| **Cell line** | IMR-90 | ATCC | Cat# CCL-186  RRID:CVCL_0347 |  |
| **Cell line** | 293T | ATCC | Cat# CRL-3216  RRID:CVCL_0063 |  |
| **Cell line** | HCT-116 | ATCC | Cat# CCL-247  RRID:CVCL_0291 |  |
| **Strain, strain background** | Female NCR-nude sp/sp (CrTac:NCr-Foxn1nu) | Taconic | NCRNU-F  RRID:IMSR_TAC:ncrnu |  |
