# Supplementary material for: STRIPAK directs PP2A activity toward MAP4K4 to promote oncogenic transformation of human cells
Source: eLife. 2020 Jan 8;9:e53003. doi: 10.7554/eLife.53003 (PMC6984821; doi:10.7554/eLife.53003)
Supplement: Transparent reporting form [file elife-53003-transrepform.pdf]

eLife's transparent reporting form

We encourage authors to provide detailed information *within their submission* to facilitate the interpretation and replication of experiments. Authors can upload supporting documentation to indicate the use of appropriate reporting guidelines for health-related research (see EQUATOR Network), life science research (see the BioSharing Information Resource), or the ARRIVE guidelines for reporting work involving animal research. Where applicable, authors should refer to any relevant reporting standards documents in this form.

If you have any questions, please consult our Journal Policies and/or contact us: editorial@elifesciences.org.

Sample-size estimation

- You should state whether an appropriate sample size was computed when the study was being designed
- You should state the statistical method of sample size computation and any required assumptions
- If no explicit power analysis was used, you should describe how you decided what sample (replicate) size (number) to use

Please outline where this information can be found within the submission (e.g., sections or figure legends), or explain why this information doesn't apply to your submission:

For each experiment, sample-size estimation was determined based on effect size, and the assays were repeated following the convention. Permutation was performed when necessary to determine the false-discovery rates.

Replicates

- You should report how often each experiment was performed
- You should include a definition of biological versus technical replication
- The data obtained should be provided and sufficient information should be provided to indicate the number of independent biological and/or technical replicates
- If you encountered any outliers, you should describe how these were handled
- Criteria for exclusion/inclusion of data should be clearly stated
- High-throughput sequence data should be uploaded before submission, with a private link for reviewers provided (these are available from both GEO and ArrayExpress)

Please outline where this information can be found within the submission (e.g., sections or figure legends), or explain why this information doesn't apply to your submission:

eLife Sciences Publications, Ltd is a limited liability non-profit non-stock corporation incorporated in the State of Delaware, USA, with company number 5030732, and is registered in the UK with company number FC030576 and branch number BR015634 at the address Westbrook Centre, Milton Road Cambridge CB4 1YG, UK | March 2019

All assays unless indicated were performed in triplicates. Soft agar experiments were performed in triplicates, and the graphs are plotted based on the mean and standard error of these experiments, as indicated in the figure legends. In vivo tumor, formation assays were performed across five individual mice injected in three sites. In vitro kinase assays were performed in three replicates, except Figure 6B, which was performed as biological replicates with two independent experiments carried out by different individuals at different time points. iTRAQ Phosphoproteomic experiments were performed as two independent biological and technical replicate experiments.
SILAC was performed as two biological replicates. MudPIT was performed once, as indicated in the figure, and the results were subsequently validated using Co-IP assays. RNAseq experiments were performed in triplicates, and both raw and processed data are available on GEO accession GSE118272. Raw mass spectrometry data files are from iTRAQ, and SILAC experiments are available for download at ftp://massive.ucsd.edu/MSV000084422/ and MudPIT experiment from Massive: ftp://massive.ucsd.edu/MSV000084662/ and ProteomeXchange:http://proteomecentral.proteomexchange.org/cgi/GetDataset?ID=PXD016628. All processed data files are provided as supplementary files.

Statistical reporting

- Statistical analysis methods should be described and justified
- Raw data should be presented in figures whenever informative to do so (typically when N per group is less than 10)
- For each experiment, you should identify the statistical tests used, exact values of N, definitions of center, methods of multiple test correction, and dispersion and precision measures (e.g., mean, median, SD, SEM, confidence intervals; and, for the major substantive results, a measure of effect size (e.g., Pearson's r, Cohen's d)
- Report exact p-values wherever possible alongside the summary statistics and 95% confidence intervals. These should be reported for all key questions and not only when the p-value is less than 0.05.

Please outline where this information can be found within the submission (e.g., sections or figure legends), or explain why this information doesn't apply to your submission:

Statistical analyses for all the graphs were performed using Graphpad Prism Software. Student's T-tests using parametric test assuming both populations with same SD were performed, and the calculated p-values are indicated in figure legends. For analysis in Fig. 7A, and Fig. 8C-E, the FDRs are computed from empirical p-values using the standard Benjamini-Hochberg procedure. The FDRs are obtained from an empirical permutation test where the target profile is randomly permuted to generate a null distribution for the Information Coefficient values.

(For large datasets, or papers with a very large number of statistical tests, you may upload a single table file with tests, Ns, etc., with reference to sections in the manuscript.)

Group allocation

- Indicate how samples were allocated into experimental groups (in the case of clinical studies, please specify allocation to treatment method); if randomization was used, please also state if restricted randomization was applied
- Indicate if masking was used during group allocation, data collection and/or data analysis

Please outline where this information can be found within the submission (e.g., sections or figure legends), or explain why this information doesn't apply to your submission:

N/A

Additional data files ("source data")

- We encourage you to upload relevant additional data files, such as numerical data that are represented as a graph in a figure, or as a summary table
- Where provided, these should be in the most useful format, and they can be uploaded as "Source data" files linked to a main figure or table
- Include model definition files including the full list of parameters used
- Include code used for data analysis (e.g., R, MatLab)
- Avoid stating that data files are "available upon request"

Please indicate the figures or tables for which source data files have been provided:

eLife Sciences Publications, Ltd is a limited liability non-profit non-stock corporation incorporated in the State of Delaware, USA, with company number 5030732, and is registered in the UK with company number FC030576 and branch number BR015634 at the address Westbrook Centre, Milton Road Cambridge CB4 1YG, UK | March 2019

Processed Proteomic data for Figures 1B, 3B, 3D, and 6B are provided as supplementary files. Source data for RNAseq analyses in Figure 7A have been uploaded to GEO, and the normalized RNAseq values and the genesets used in Figures 7A and 8C analyses are provided in the supplementary files. Source data, which include the quantification used to plot graphs for the figures, are provided with the manuscript.

eLife Sciences Publications, Ltd is a limited liability non-profit non-stock corporation incorporated in the State of Delaware, USA, with company number 5030732, and is registered in the UK with company number FC030576 and branch number BR015634 at the address Westbrook Centre, Milton Road Cambridge CB4 1YG, UK | March 2019
